# Innovative Rhizosphere-Based Enrichment under P-Limitation Selects for Bacterial Isolates with High-Performance P-Solubilizing Traits

**DOI:** 10.1128/spectrum.02052-22

**Published:** 2022-10-11

**Authors:** Noémie De Zutter, Maarten Ameye, Pieter Vermeir, Jan Verwaeren, Leen De Gelder, Kris Audenaert

**Affiliations:** a Laboratory of Applied Mycology and Phenomics (LAMP), Department of Plants and Crops, Faculty of Bioscience Engineering, Ghent University, Ghent, Belgium; b Laboratory of Environmental Biotechnology, Department of Biotechnology, Faculty of Bioscience Engineering, Ghent University, Ghent, Belgium; c Inagro, Rumbeke-Beitem, Belgium; d Laboratory of Chemical Analysis (LCA), Faculty of Bioscience Engineering, Ghent University, Ghent, Belgium; e Research Unit Knowledge-based Systems (KERMIT), Department of Data Analysis and Mathematical Modelling, Ghent University, Ghent, Belgium; University of Minnesota

**Keywords:** biostimulants, maize, phosphate solubilizing bacteria, plant growth promotion, rhizosphere competence

## Abstract

The use of phosphate solubilizing bacteria (PSB) as inoculants for the rhizosphere is a well-known strategy to mitigate P-deficiency in plants. However, despite the multiple modes of action to render P available for plants, PSB often fail to deliver in the field as their selection is often based on a single P-solubilizing trait assessed *in vitro.* Anticipating these shortcomings, we screened 250 isolates originating from rhizosphere-based enriched consortia for the main *in vitro* P-solubilizing traits, and subsequently grouped the isolates through trait-based HCPC (hierarchical clustering on principal components). Representative isolates of each cluster were tested in an *in planta* experiment to compare their *in vitro* P-solubilizing traits with their *in planta* performance under conditions of P-deprivation. Our data convincingly show that bacterial consortia capable to mitigate P-deficiency *in planta* were enriched in bacterial isolates that had multiple P-solubilizing traits *in vitro* and that had the capacity to mitigate plant P-stress *in planta* under P-deprived conditions. Furthermore, although it was assumed that bacteria that looked promising *in vitro* would also have a positive effect *in planta*, our data show that this was not always the case. Opposite, lack of performance *in vitro* did not automatically result in a lack of performance *in planta*. These results corroborate the strength of the previously described *in planta*-based enrichment and selection technique for the isolation of highly efficient rhizosphere competent PSB.

**IMPORTANCE** With the growing awareness on the ecological impact of chemical phosphate fertilizers, research concerning the use of phosphate solubilizing bacteria (PSB) as a sustainable alternative for, or addition to these fertilizers is of paramount importance. In previous research, we successfully implemented a plant-based enrichment technique for PSB, which simultaneously selected for the rhizosphere competence and phosphate solubilizing characteristics of bacterial suspensions. Current research follows up on our previous findings, whereas we screened 250 rhizobacteria for their P-solubilizing traits and were able to substantiate the results obtained from the enriched suspensions at a single-isolate level. With this research, we aim for a paradigm shift toward the plant-based selection of PSB, which is a more holistic approach compared to the plate-based methods. We emphasize the strength of the previously described plant-based enrichment and selection technique for the isolation of highly efficient and diverse PSB.

## INTRODUCTION

Given the environmental risk of chemical phosphorus (P) fertilizers, research, and industry now turn to a sustainable approach for managing P-deficiency in agricultural soils. P is an essential macronutrient for plant growth and development, as it is involved in the regulation of several metabolic pathways and is a component of key molecules such as nucleic acids, phospholipids, and ATP (1, 2). The P-bioavailability for plants is determined by the presence of orthophosphates (H_2_PO_4_- and HPO_4_^2−^) in the soil solution. However, these orthophosphates are prone to a rapid immobilization through adsorption onto clay minerals, precipitation into various salts or fixation into organic P (3). To meet a crop’s P-requirements, (chemical) P-fertilizers derived from rock phosphate are widely applied on agricultural soils. Although applied in excess of the crop’s growing needs, the majority of the readily available P is rapidly immobilized into organic P or calcium-, aluminum- or iron phosphates, again rendering it inaccessible for plants (4). Additionally, the use of chemical P-fertilizers causes severe environmental risks by impacting the soil health and disturbing the soil microbial diversity. Finally, the over-application of P-fertilizers causes surface runoff and leaching of excessive P toward waterbodies, resulting in the eutrophication of surface waters (5, 6). On top of this, rock phosphate is a nonrenewable resource gained through highly polluting mining and processing procedures (5, 7). In this regard, microbial P-solubilization in the rhizosphere of plants is an important biostimulatory trait and the use of phosphate solubilizing bacteria (PSB) is on the rise.

### Direct modes of action for P-solubilization.

Microorganisms depend on 2 major strategies to render P available for their own growth, and thereby also for the plants which rhizosphere they inhabit: (i) inorganic P-solubilization through the exudation of (in)organic acids, the secretion of siderophores or the production of EPS; and (ii) organic P-solubilization through enzymatic breakdown (2, 8, 9). The predominant mechanisms of P-solubilization are through organic acid production, H^+^-excretion and acid phosphatases (2, 4, 10–12).

### Inorganic P-solubilization.

A first way to solubilize inorganic phosphate is through the exudation of low molecular weight organic acids (OAs) such as citric acid, malic acid, oxalic acid, and gluconic acid. By secreting these OAs, microorganisms cause a local decrease in pH, which results in the increased solubility of calcium phosphates (13). Under increased acidity, protons compete with metal ions such as Fe^3+^ and Al^3+^ for binding sites, hence releasing P from these metal-complexes. Similarly, acidic anions may compete with PO_4_^3−^, consequently resulting in the release of phosphates (2).

Bacterial production of inorganic acids such as sulfuric-, nitric-, hydrochloric- and carbonic acid contribute to a lesser extent to the inorganic P-solubilization (2). On the other hand, bacterial H_2_S-production has emerged as an important P-solubilizing trait, through the reaction of H_2_S with iron phosphate, which results in the production of ferrous sulfate and the release of phosphate (14).

Furthermore, inorganic P-solubilization can be mediated through the excretion of metal-chelating compounds (15, 16). Specifically, siderophores are high affinity iron chelating compounds that can be produced by bacteria under iron-deficiency, thereby promoting solubilization of the Fe-P complexes in soil (15, 17). In addition to siderophores, exopolysaccharides (EPS) involved in bacterial biofilm formation (18) can form complexes with metal ions (e.g., iron, aluminum and calcium) present in soils and are thus capable of making metal bound P available for biological processes in both plants and soil microorganisms (2).

### Organic P-solubilization.

The amount of organic P in the total soil P pool ranges between 4 and 90% (19). Organic phosphorus predominantly occurs in soils as phosphomonoesters (i.e., phytate, lower-order inositol phosphates, and other sugar phosphates), phosphodiesters (i.e., phospholipids and nucleic acids), and a large pool of poorly characterized monoester compounds (12, 20). Due to the resistance of these compounds to chemical hydrolysis, bioconversion of organic P is paramount to render it bioavailable for plants. This bioconversion occurs by means of enzymatic mineralization, in particular through nonspecific acid phosphatases (NSAPs), phytases, phosphonatases and C-P lyases (17). The main enzymatic activity can be attributed to phytases and NSAPs, because of the foremost presence of their respective substrates in soil (21).

### Indirect contribution of microorganisms to plant P-uptake.

As opposed to the direct solubilization of phosphate, the role of microbial biomass turnover contributes to the indirect release of phosphates (2, 8). Since P is an essential component for cellular functions, microorganisms will accumulate this P in microbial biomass in the first place (22). These microorganisms serve as P-sinks, and become a source of P to plants upon P-release from their cells (5).

Finally, the microbial production of phytohormones and the ability of microorganisms to modify hormone signaling in plants contribute to plant growth and root development (23). For example, microbial auxin and cytokinin production alters the root system architecture by enhancing root growth and root hair formation (24). By optimizing the root architecture, water, and nutrient assimilation can be improved through an increased contact surface area between the roots and soil solution and through a more precise placement of roots in nutrient-rich zones in the soil (25).

### PSB: toward selection and application.

The most commonly adopted selection method starts with an extensive *in vitro* screening of isolates originating from e.g., rhizosphere microbial communities, after which the best performing isolates are selected to evaluate their effectiveness in plant trials (26–29). The success and failure of these PSB is not only dependent on their phosphate solubilizing capacity, but also on their rhizosphere competence, their ability to compete with the innate microbiome and to persist in the physicochemical conditions of the intended farmlands (30–33). Therefore, the *in vitro* success of an isolate does not guarantee its *in planta* effectiveness, which is one of the main reasons why these PSB often fail to deliver in practice.

Some studies take the rhizosphere competence and concomitant *in planta* effectiveness of bacterial isolates into account by incorporating aspects of the rhizosphere environment, either during *in vitro* selection (e.g., the use of root exudates as a sole carbon source [34]) or through *in planta* selection of bacterial isolates (35–38). Additionally, over the past decades, bacterial gene-expression during plant-bacterial interactions in the rhizosphere have been studied and reviewed, especially for Pseudomonas spp. The ability of bacterial species to thrive in the rhizosphere is not attributed to one unique genetic trait, but relies on diverse cellular pathways and physiological responses (39–41). In previous research, we optimized an iterative *in planta* method to enrich the maize rhizosphere for bacteria that have both the rhizosphere competence and the P-solubilization trait (38). Starting from a native plant rhizosphere, the bacterial community was passed through 4 consecutive *in planta* enrichment steps, in which maize plants were grown under P-deficiency. After 21 days of maize growth, the rhizosphere microbial community was used as an inoculum for the next enrichment step, thereby resulting in 4 consecutive enriched cultures. In a comparative test, the culture that had been obtained after 3 consecutive enrichments proved to be capable of alleviating plant P-stress in maize plants. However, further enrichment resulted in a functional relapse of the enriched bacterial suspensions. Through a metabarcoding approach, the bacterial composition of these consortia was uncovered and the (dis)functionality of these consortia could be attributed to certain bacterial families.

In the present research, the bacterial cultures obtained through *in planta* selection under P-deficiency were used as a starting point for the *in vitro* selection of bacterial isolates with traits related to P-solubilization (Fig. S1). These consortia, and especially the functional bacterial consortium obtained after 3 enrichment steps, were considered to be valuable sources for the isolation of highly efficient, rhizosphere competent PSB.

The aims of this study were (i) to identify the *in vitro* modes of action of bacteria isolated from enriched rhizosphere bacterial suspensions of plants grown under P-limitations; (ii) to link the *in vitro* modes-of-action of these isolates to the *in planta* effectiveness of the respective enriched bacterial suspensions of which they were isolated; (iii) to evaluate whether the *in planta* enrichment procedure selected for isolates with specific *in vitro* P-solubilizing traits and/or isolates with a high efficiency for a specific *in vitro* P-solubilizing trait; and (iv) to evaluate whether the specific P-trait-related fingerprints of single bacterial isolates could be coupled to an effect of those isolates on plant P-uptake in the presence of an insoluble P-source.

## RESULTS

### P-solubilizing capacity of bacterial isolates: quantitative and categorical assessment.

**FIG 1:**
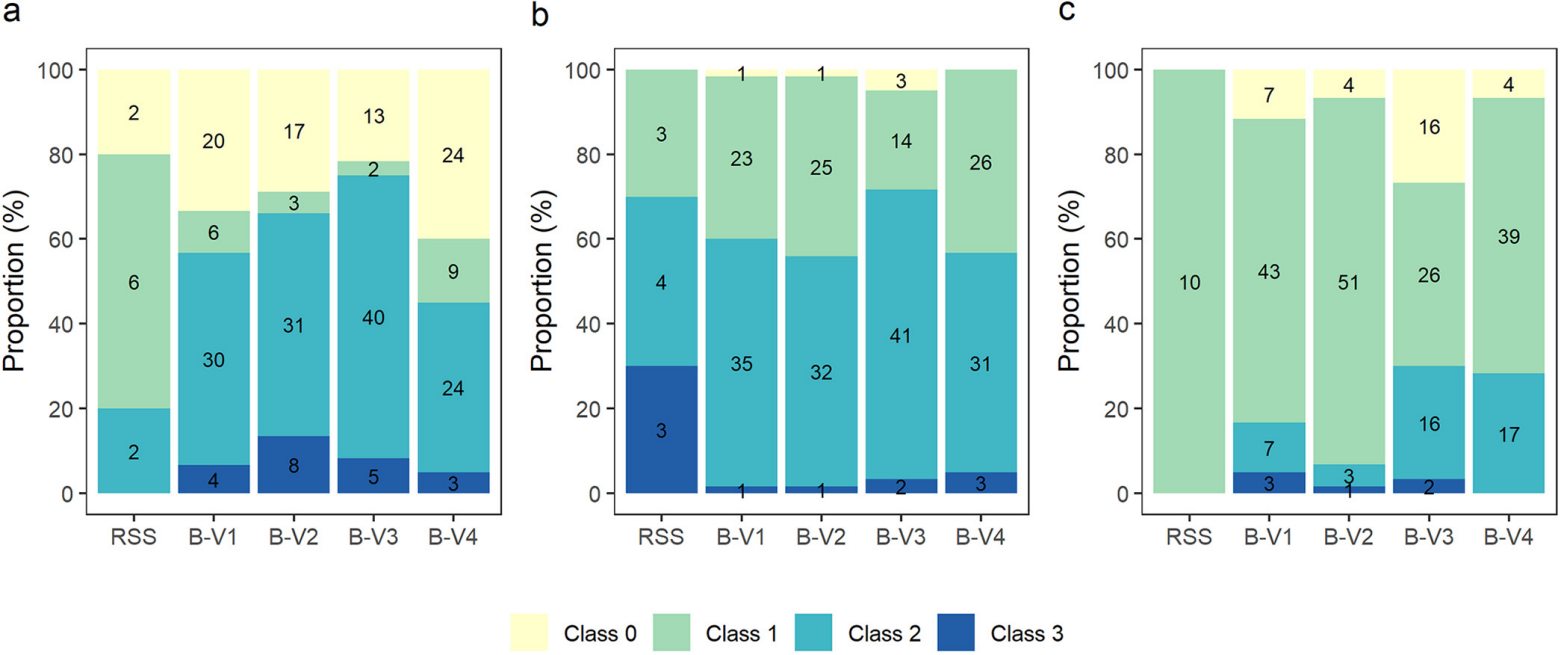
Bacterial growth of 249 isolates per group (RSS, B-V1, B-V2, B-V3, B-V4), evaluated as the average colony diameter per isolate (*n* = 3) on NBRIP supplemented with (a) iron phosphate, (b) aluminum phosphate and (c) tri-calcium phosphate. Colony diameters ≤1mm were attributed to the point inoculation and were not evaluated as true growth (Class 0). Classes were chosen based on equal intervals of colony diameters (mm): (a) Class 1] 1-1.67], Class 2] 1.67-2.33], Class 3] 2.33-3]; (b) Class 1] 1-2.5], Class 2] 2.5-4], Class 3] 4-6]; (c) Class 1] 1-4], Class 2] 4-7], Class 3] 7-10].

A total of 249 bacterial isolates, randomly selected from the rhizosphere start suspension (RSS, 10 isolates), or from the enriched bacterial suspensions (B-V1 to B-V4, 60 isolates per suspension), were screened for their P-solubilizing capacity. In brief, the RSS was obtained by sampling the maize rhizosphere from different locations in Flanders. This RSS was used as the starting point in an *in planta* platform developed to enrich for bacterial strains under selective pressure for their rhizosphere competence and P-solubilizing capacity of aluminum and iron phosphate. For more details on the origin of the bacterial consortia, we kindly refer to De Zutter et al. (38) and Fig. S1. Bacterial growth of the isolates on solid medium supplemented with either tri-calcium (TCP), aluminum (AlP) or iron (FeP) phosphate was monitored by measuring the bacterial colony diameter. Per P-source, diameters were divided into equal classes and the distributions were evaluated (Fig. 1a to c). In order to have a valid comparison, we only considered the enriched bacterial suspensions (B-V1 to B-V4) for statistical analysis. The RSS, which was used as a starting point for the *in planta* enrichment but not yet subjected to the enrichment platform itself, was omitted from the statistical analysis. Based on the visual assessment of the data, a shift in frequency distribution between no growth (Class 0) and growth (Class 1–3) can be observed for NBRIP supplemented with FeP and TCP (Fig. 1a and c), while no clear trend can be observed for NBRIP supplemented with AlP (Fig. 1b). On one hand, a non-significant enrichment for isolates able to grow on NBRIP-FeP was observed up until B-V3 (B-V1 to B-V3 resp. B-V2 to B-V3, *P* = 0.17 resp. 0.465), after which a relapse occurred in B-V4 compared to the B-V3 (*P* = 0.030). On the other hand, an adverse effect was observed for bacterial growth on NBRIP-TCP, where a decrease in isolates able to grow on the medium was observed from B-V1 and B-V2 to B-V3 (*P* = 0.037 resp. 0.004), and an increase from B-V3 to B-V4 (*P* = 0.003). Subsequently, the frequency distributions of the different growth classes were evaluated for each P-source. Significant differences were observed between B-V3 and B-V4 for NBRIP-FeP and NBRIP-AlP, respectively (*P* = 0.007, 0.042, respectively), where in both cases, more isolates of B-V3 display a growth in class 2 compared to B-V4.

All isolates were evaluated for their P-solubilizing capacity in liquid NBRIP-medium supplemented with iron- and aluminum phosphate against an uninoculated blank (Fig. S2). The top 20 isolates, capable of solubilizing the highest amount of P, were selected and their distribution among the bacterial consortia was evaluated. In case of an equal distribution of high performing isolates among the consortia, the expected ratio would be 4% in the RSS and 24% in B-V1 to B-V4. However, the observed ratios in the RSS, B-V1, B-V2, B-V3 and B-V4 are 0%, 15%, 30%, 35% and 20% respectively. The data show that there was a non-significant (*P* = 0.342) increased proportion of high performing isolates in both B-V2 and B-V3. Additionally, the individual iron- and aluminum phosphate solubilizing capacity of a random subset of isolates was evaluated (data not shown). In this experiment, bacterial isolates were more capable of solubilizing iron phosphate, with a maximum P-solubilization rate of 459 ± 187 mg P.L^−1^ (mean ± sd.), compared to aluminum phosphate (maximum P-solubilization rate of 47 ± 13 mg P.L^−1^) (mean ± sd.).

### Evaluation of potential bacterial modes of action for P-solubilization.

**FIG 2:**
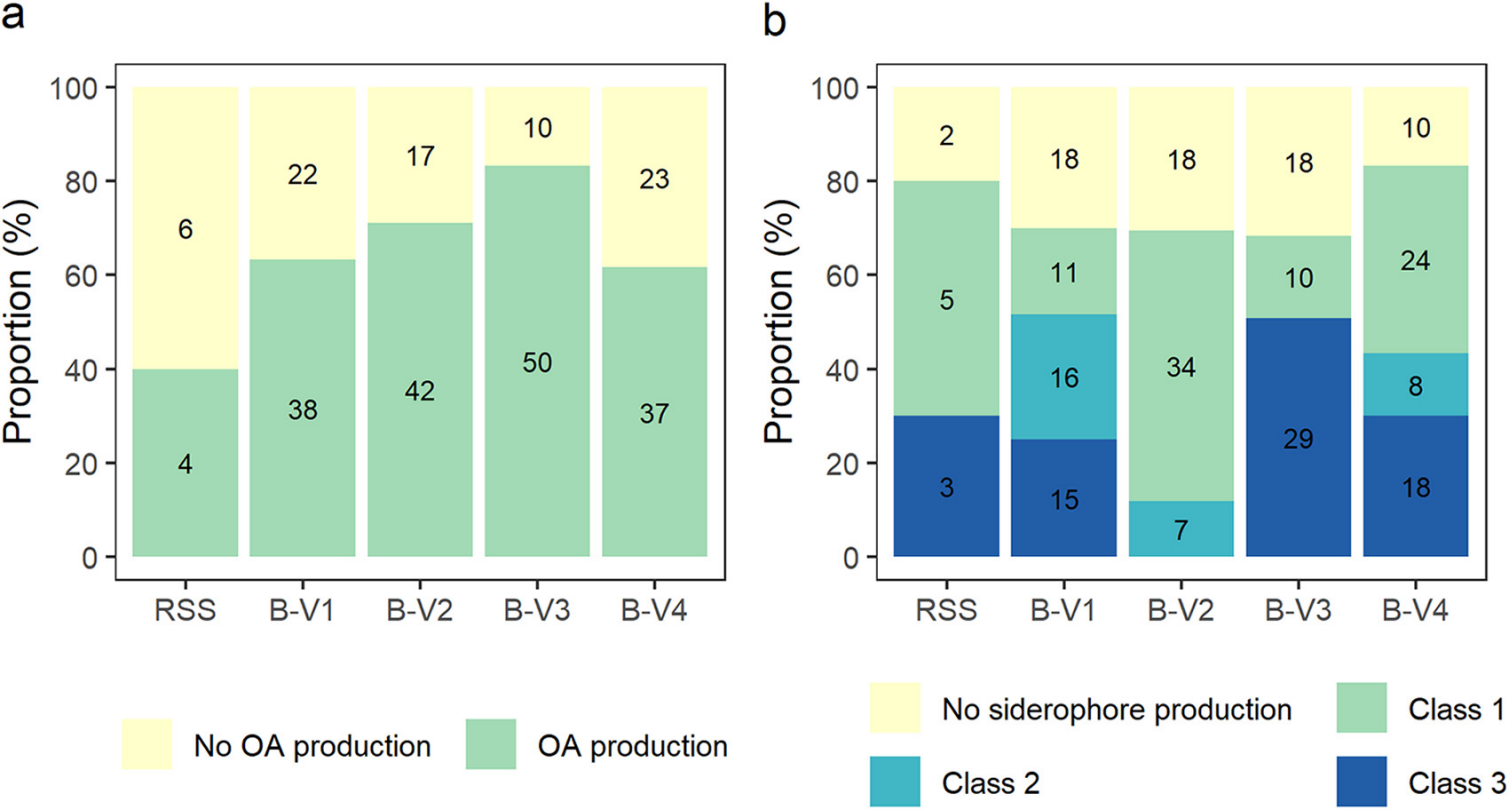
Proportion of bacterial isolates per group (RSS, B-V1, B-V2, B-V3, B-V4) that were capable of producing (a) organic acids on MPVK-agar supplemented with iron- and aluminum phosphate and (b) siderophores (Class 1: small halo, Class 2: moderate halo, Class 3: large halo) on CAS-agar.

The bacterial production of organic acids (OAs) was evaluated on MPVK-agar supplemented with iron- and aluminum phosphate in a 1:1 mol ratio. OA production is hallmarked by a yellow discoloration of the medium (pH < 4.6) and was scored by its presence or absence (Fig. 2a). The frequency of OA producing bacteria increased over the enriched consortia and reached a maximum of 83.3% in B-V3, which was a significant improvement over B-V1 (63.3%; *P* = 0.013). However, a significant decrease of 21.6% of OA producing bacteria was observed in B-V4 when comparing with B-V3 (*P* = 0.008).

Additionally, bacterial siderophore production was ordinally evaluated on CAS-agar (Fig. 2b and Fig. S3). In a first assessment, the frequency of siderophore producers and non-producers was evaluated over the different consortia. The majority of all isolates (73.1%) secreted siderophores, and these siderophore producing bacteria were evenly distributed among the different consortia. In a second assessment the quantity of the produced siderophores was included based on an ordinal scoring system (Table S3). The frequency of high-producing (class 3) isolates proved to be the highest in B-V3, while the frequency of low producing (class 1) isolates was the highest in B-V2.

Finally, bacterial H_2_S-production was evaluated in SIM-medium. Only 6% of all isolates were able to produce H_2_S *in vitro*, and the distribution was equal among the different groups.

### Factor and clustering analysis of numeric and categorical data.

To get a holistic view on the P-solubilizing capacity and potential modes of action of the isolates, all traits were converted to a numeric score between 0 (no growth/production) and 1 (highest growth/production). A ranking was made based on the combined score of these traits and the distribution of isolates in the top 50 was evaluated (Table S4). The frequency distributions of the bacterial consortia in this top 50 were 10%, 6%, 52% and 32% for the B-V1, B-V2, B-V3, and B-V4 respectively, which differs significantly from the expected ratio in case of an equal distribution among the consortia (*P* = 0.003).

**FIG 3:**
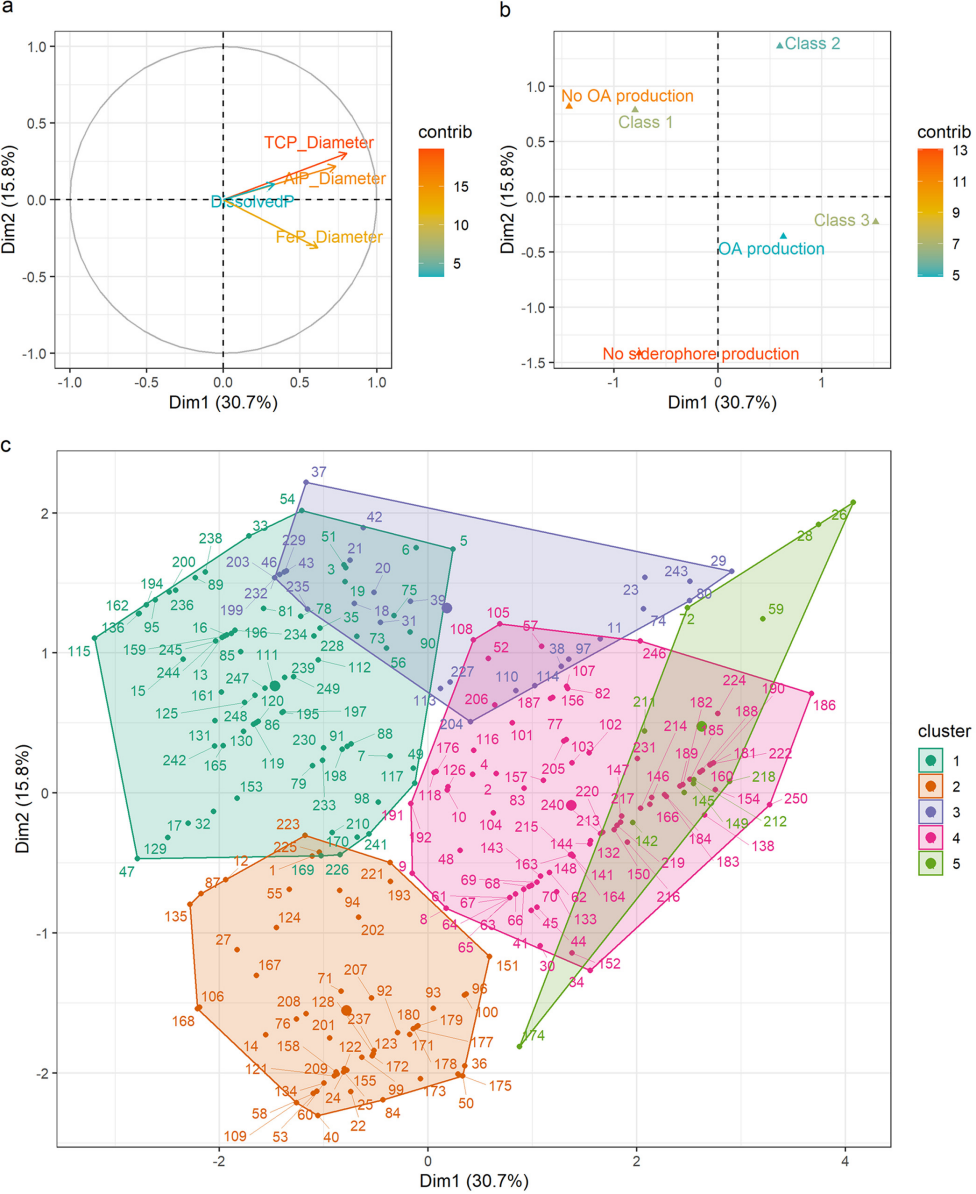
Factor maps for the first two dimensions of (a) the numeric variables and (b) the nominal variables obtained from factor analysis of mixed data (FAMD), and (c) individual scores obtained through factor analysis of mixed data (FAMD) in the first 2 dimensions. The analysis were conducted on the total data set of 249 bacterial isolates. Clustering occurred by means of hierarchical clustering of principal components (HCPC). Cluster 1: *n* = 70; Cluster 2: *n* = 56; Cluster 3: *n* = 27; Cluster 4: *n* = 82; Cluster 5: *n* = 11.

The combined numeric and nominal data were explored using a factor analysis of mixed data (FAMD), which explained 46.5% of the total variance in the first 2 dimensions (30.7% and 15.8% respectively [Fig. S4]). Numeric variables that correlate the most with the first dimension are bacterial growth on NBRIP supplemented with TCP, AlP, and FeP (0.804, 0.731, and 0.616, correlation, respectively) (Fig. 3a). Individuals with a negative value for the first dimension depict bacterial isolates with poor siderophore and OA producing abilities, while those with a positive correlation display high siderophore and OA producing abilities (Fig. 3b). In short, the first dimension of FAMD represents a gradient of isolates that show poor quantitative P-solubilizing capacities and few potential P-solubilizing modes of action (left) to possibly highly performing PSB (right).

Through hierarchical clustering on principal components (HCPC), five clusters were identified and plotted in a 2D-graph (Fig. 3c). Cluster 1 (*n* = 70) was characterized by bacteria with below average growth on NBRIP supplemented with all 3 P-sources (iron, aluminum, and calcium phosphate) that displayed no organic acid production and class 1 siderophore production. The bacteria in cluster 2 (*n* = 56) were mainly characterized by below average growth (compared to the overall mean) on NBRIP supplemented with aluminum and calcium phosphate, and the lack of siderophore producing abilities. Cluster 3 (*n* = 27) was characterized by bacteria that display class 2 siderophore production. Bacteria belonging to cluster 4 (*n* = 82) were hallmarked by an above average growth on NBRIP-agar with all 3 P-sources, and were likely to display both organic acid production and class 3 siderophore production. Finally, cluster 5 (*n* = 11) contained bacteria that were able to solubilize P in liquid medium at a high rate. These bacteria also produced organic acids and class 2 or 3 siderophores (Table S5 and S6).

### Identification of bacterial isolates through 16S rRNA gene.

**FIG 4:**
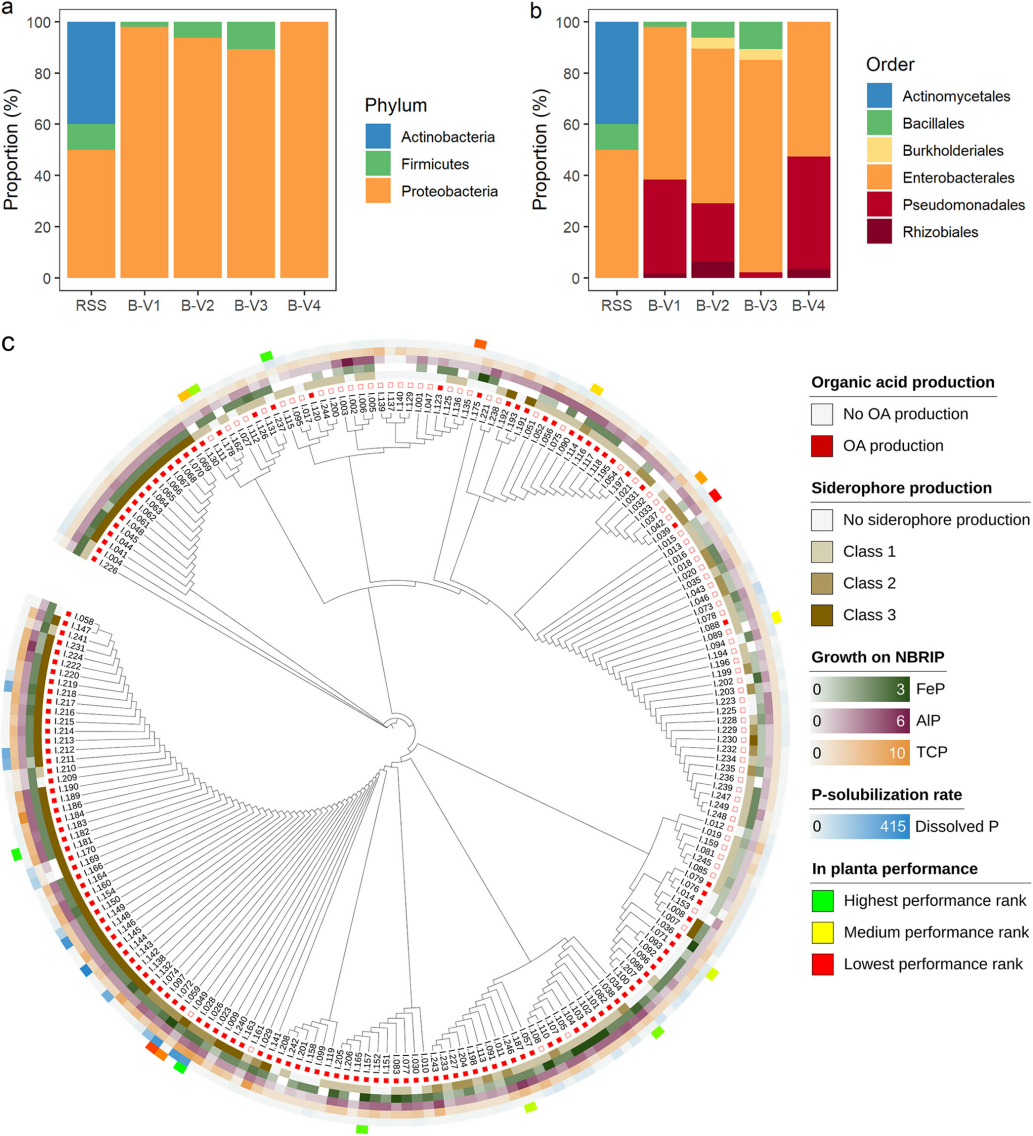
(a) and (b) Classification of bacterial isolates per group (RSS, *n* = 10; B-V1, *n* = 53; B-V2, *n* = 48; B-V3, *n* = 47; B-V4, *n* = 58) based on 16S rRNA gene per (a) phylum and (b) order. (c) Phylogenetic cladogram inferred from the 16S rRNA gene sequences, displaying the phylogenetic relationships between the bacterial isolates. Annotation layers were added based on the *in vitro* traits (i.e., organic acid production, siderophore production, growth on NBRIP supplemented with FeP, AlP and TCP and P-solubilization in liquid medium) of all isolates, and the *in planta* performance of a subset of the isolates.

All isolates were further identified by their 16S rRNA gene (Fig. 4 and Table S7). The majority of all isolates belonged to the phylum of the *Proteobacteria* (*n* = 200, 80.3%), and more specifically to the orders of the *Enterobacterales* (*n* = 134, 53.8%) and *Pseudomonadales* (*n* = 56, 22.5%). Only 4.0% (*n* = 10) of all isolates belonged to the order of the *Bacillales* (*Bacillus* spp. and *Paenibacillus* spp.). The frequency distribution into phyla differed significantly between the RSS and the enriched consortia (*P* < 0.001 for all), and between B-V4 and the other consortia (B-V1, B-V2 and B-V3; *P* = 0.006, 0.003 respectively, <0.001) (Fig. 4a). At a higher taxonomic level, frequency distributions of the bacterial orders significantly differed between all enriched consortia, except between B-V1 and B-V2, and B-V1 and B-V4. Interestingly, B-V3 was hallmarked by the reduced presence of *Pseudomonadales* (1.7%), the absence of *Rhizobiales* and the highest abundance of *Bacillales* (8.3%) (Fig. 4b and Table S8). The abundance of these bacterial orders increased (decreased for *Bacillales*) again after the next enrichment cycle, in B-V4. Finally, the bacterial identifications were linked to the clusters obtained through HCPC (Table S9). Clusters 1 and 3 were dominated by *Pseudomonadales* (41.4% resp. 55.6%), while clusters 4 and 5 were dominated by the *Enterobacterales* (89.0% and 90.9%, respectively).

The taxonomic distribution of the bacterial traits was visualized by means of a phylogenetic cladogram (Fig. 4c). While the production of organic acids and siderophores appeared to be well-conserved phylogenetic traits dominantly perceived in *Pantoea* spp. and Pseudomonas spp., the ability of bacteria to grow on solid medium supplemented with FeP, AlP, and TCP showed a more scattered, less conserved distribution among the phylogenetic clusters.

### *In planta* evaluation of a diverse subset of bacterial isolates selected based on FAMD and HCPC.

**FIG 5:**
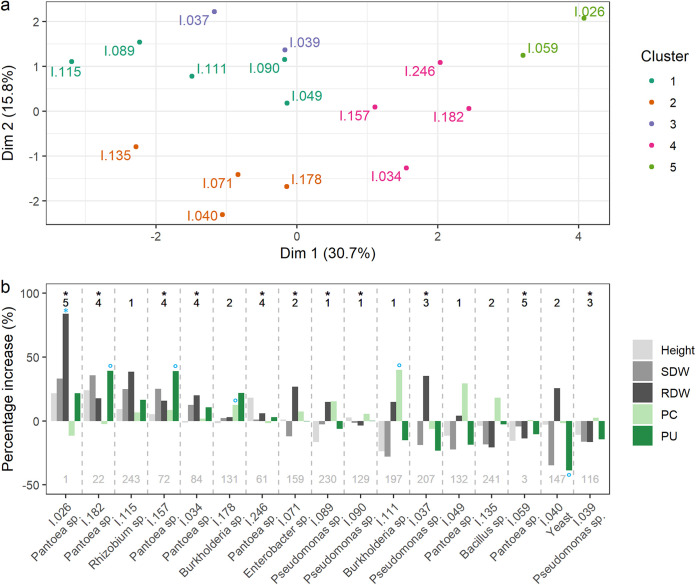
(a) Representation of the subset of bacterial isolates evaluated for their *in planta* growth-promoting and P-solubilizing traits; (b) Effect of the subset of bacterial isolates on plant growth promotion (height, shoot dry weight and root dry weight; gray) and on phosphate content (PC) -and uptake (PU) (green), represented as the percentage increase compared to the untreated control plants. Significant increases or decreases compared to the untreated control plants are indicated in blue (*, *P* < 0.05; ^o^, *P* < 0.1). The respective cluster in which the isolate was divided is indicated on top. The presence of the isolates in the rhizosphere of the maize plants was evaluated at the end of the experiment and is indicated by the presence of an asterisk. The *in vitro* ranking of the isolates is indicated below the bars in gray.

Based on the factor and clustering analysis of all data, a selection of 17 bacterial isolates was made to test their plant growth-promoting capacity and impact on plant P-content (mg P per kg dried plant material) and P-uptake (mg P per plant). The isolates were chosen over the different clusters, based on their differential *in vitro* capacities (Fig. 5a). The percentage increase (or decrease) in plant height, shoot, and root dry weights, plant P-content and P-uptake were calculated per treatment as opposed to the untreated control (Fig. 5b). Isolates I.026, I.182, and I.246 proved to promote maize growth and P-uptake, however they had an adverse effect on maize P-content. This indicates that the effect on plant P-uptake was less related to the P-solubilizing abilities of these isolates than to their ability to improve the shoot biomass, for example through the production of plant growth regulators such as auxin, cytokinin, gibberellin, or through the effect of ACC-deaminase. Another hypothesis can be that, due to the improved plant P-uptake, the plant growth was improved. Isolate I.178 had no effect on plant height nor biomass, however consistently improved the plant’s P-content and P-uptake, indicating that the effect was related to the P-solubilizing capacity of this isolate, despite the lack of growth promotion. Isolates I.089, I.111, I.049, and I.135 had an adverse effect on plant growth and shoot biomass, however improved the plant P-content. Isolates I.115 and I.157 improved plant height, dry weight, and P-uptake in maize, as well as its P-content, demonstrating both the growth-promoting and P-solubilizing capacities of these isolates. The isolates were ranked based on their overall score, from the highest combined effect on plant health parameters, to the lowest effect and were compared to their *in vitro* effectiveness (Fig. 4c). The cluster distribution in the top-6 of the best performing isolates significantly differed from the estimated distribution in case of an equal distribution (*P* < 0.001). Cluster 4 was predominantly present in the top-6 of the best performing isolates, with a prevalence of 50% (Fig. 5b). Finally, the rhizosphere colonization was evaluated at the end of the experiment. A dilution series of the rhizosphere was plated on LB-agar, after which 30 single colonies were selected per treatment and subjected to colony PCR targeting the 16S rRNA genes. The rhizosphere suspension of the untreated control plants was included to evaluate the baseline of ‘background’ bacteria, and was dominated by Pseudomonas sp. (100%). Although the PCR identification of the colonies did not allow a distinction on an isolate level, the added bacterial genera could be retrieved from the rhizosphere in 11 out of the 17 treatments (Fig. 5b and Table S10).

## DISCUSSION

The use of P-solubilizing bacteria to improve plant growth and health as an alternative to the use of chemical fertilizers has gained more research interest over the past few decades. In this study, we evaluated the effect of 249 potential phosphate solubilizing isolates obtained from several enriched consortia (38) for their *in vitro* P-solubilizing characteristics as described in the material and methods section. Hence, we investigated whether the *in vitro* characteristics of these single strains could shed light on the P-solubilizing effect of the respective consortium (BV1-BV4) they were isolated from.

For several characteristics, isolates originating from the B-V3 consortium scored generally better (i.e., more isolates with P-solubilizing traits or a higher efficiency in the particular traits) than isolates from other consortia (B-V1, B-V2 and B-V4). In this research, bacterial P-solubilization in liquid medium reached high rates compared to data described in the existing literature, especially for iron phosphate solubilization (42, 43). One possible reason for these low P-solubilizing rates in literature is that the majority of research focuses on the solubilization of TCP as an insoluble P-source. Results showed that TCP was not an appropriate P-source for isolate selection from the consortia used in this research. As these bacterial isolates originated from the rhizosphere of plants grown under the selection of iron- and aluminum phosphate as insoluble P-source, it was not surprising that higher P-solubilizing rates for iron phosphate were observed. These results confirm previous conclusions by Bashan et al. (44) and De Zutter et al. (9) on the importance of using a P-source representative for the intended soil at the target location during *in vitro* screenings of bacterial isolates. Additionally, bacterial H_2_S production was not deemed as a representative trait for a consortium’s P-solubilizing capacity. An explanation may also be found in the origin of the microbial consortia from which the isolates were selected. As these consortia were obtained from the rhizosphere of maize plants grown in a sand-polymer substrate, the occurrence of strict anaerobic circumstances was unlikely. As bacterial sulfur reduction occurs under anaerobic circumstances (5), i.e., in paddy soils, bacteria with the trait of sulfur reduction might be out-selected in the consortia by other microorganisms.

**FIG 6:**
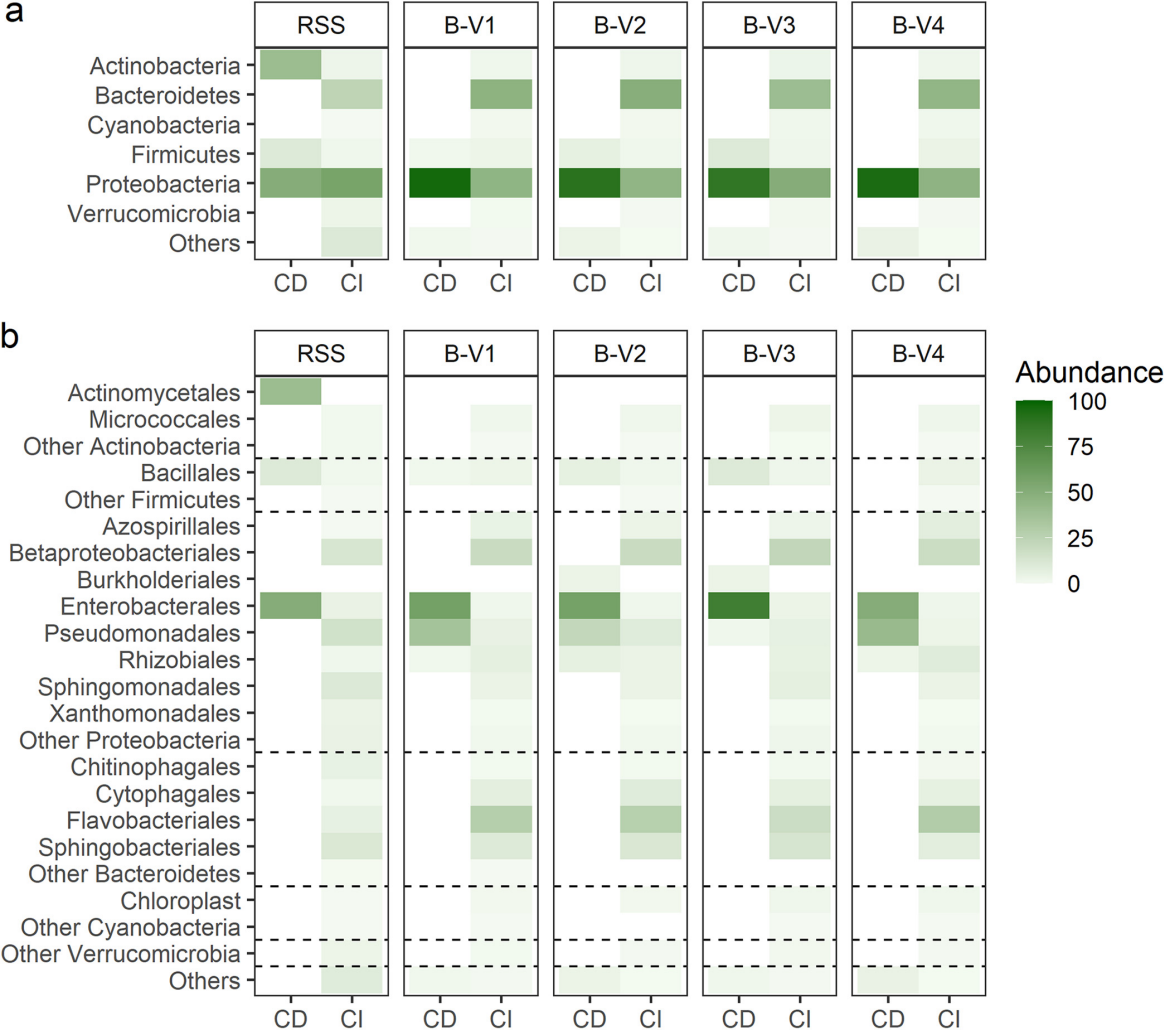
Comparison between the bacterial identities obtained through a cultivation-dependent (CD) and cultivation-independent (CI) method at the taxonomic level of (a) phylum and (b) order. The term ‘*Betaproteobacteriales’* was used in the culture independent analysis as an umbrella order for the *Burkholderiales* (87.3%), *Nitrosomonadales* (10.0%), *Neisseriales* (1.8%), *Rhodocyclales* (0.7%) and *Procabacteriales* (0.2%).

Subsequently, the bacterial identities obtained through cultivation-dependent methods in the present paper were compared to the results obtained through the cultivation independent method, i.e., metagenomics, in prior research (38). While the omnipresence of the *Proteobacteria* was detected through both techniques, the presence of *Bacteroidetes* could only be detected through the cultivation independent method (Fig. 6a). At a higher taxonomic level, it became apparent that the diversity within the bacterial phyla differed greatly between the cultivation-dependent and independent method (Fig. 6b). Of the 32 orders identified through metagenomics, only 7 were recovered in the cultivation-dependent isolation. This higher diversity in the cultivation independent method might be attributed to the important role that is attributed to low molecular weight exudates that are absent in the cultivation-dependent method. In a study using *Pinus radiata*, it was shown that model exudate solutions based on low molecular weight organic acids (quinic, lactic, maleic acids) and sugars (glucose, sucrose, fructose) increased bacterial taxon richness compared to control soils (45). In a review by Korenblum et al. (2022), it was shown that root exudates, including secondary metabolites such as benzoxazinoids, coumarins, flavonoids, indolic compounds and terpenes, shape the rhizosphere microbiome. These inter-kingdom chemical interactions comprise the consumption, reuse, and re-designation of metabolites. This review also coined the term “Systemically Induced Root Exudation of Metabolites” in which the rhizosphere microbiome governs plant metabolism by inducing systemic responses that shift the metabolic profiles of root exudates. This highlights the importance of root exudates in shaping the complexity of a root microbiome (46). Finally these root exudates also play a part in biofilm formation once the bacteria have reached the roots (47). As all these interactions are absent in a plating assay, we assume that this specific rhizosphere flora was overgrown by fast growing copiotrophs in the plating assays leading to a lower diversity compared to the *in planta* approach. Remarkably, in both cultivation-dependent and independent methods, the order of the *Bacillales* was only present in low abundances compared to other bacterial orders. Although *Bacillus* spp. have a proven efficacy as PSB (9), they appear to be outcompeted by other bacterial species during consecutive *in planta* enrichment.

At the level of single isolates, literature comparing the *in vitro* and *in planta* efficiency of an isolate is ubiquitous. The most adopted selection strategy for PSB is a top-down strategy, in which large scale *in vitro* screenings are followed by small scale greenhouse trials with the top-of-the-class isolates. As stated by Collavino et al. (48), the ability of a bacterial isolate to solubilize P *in vitro* is not necessarily associated with its ability to promote plant growth or improve a plant’s P-status. This was confirmed in our research, where isolate I.059, identified as *Pantoea* sp., was among the best performing isolates *in vitro*, but failed to alleviate P-stress in a plant, while other strains that did not perform well *in vitro*, performed well under P-limiting conditions *in planta*. The effectiveness of a bacterial isolate in association with a host plant is not only dependent on its *in vitro* biostimulatory traits, but also on its root colonizing abilities (49).

Based on the overall ranking of the *in vitro* performance of the isolates (Table S4), the top 50 was dominated by isolates originating from B-V3 (52%), which was the bacterial consortium capable of alleviating P-stress the best in maize plants as reported in our previous study (38). These results substantiate the effectiveness of an *in planta*-based enrichment and selection technique for bacteria with P-solubilizing activity, as microorganisms that either harbor a specific trait (i.e., growth on NBRIP supplemented with iron phosphate and organic acid production; Fig. 1a and Fig. 2a), or that are more efficient for a specific trait (i.e., growth on NBRIP supplemented with aluminum phosphate and siderophore production; Fig. 1b and Fig. 2b), were enriched in the consortium that proved to be efficient *in planta* (B-V3) and were therefore picked up in the analysis. Finally, the functional relapse which was observed in B-V4 in our previous research (38) was also detected in the *in vitro* approach, where in general, isolates from B-V4 performed worse than isolates from B-V3. The presence of alleged cross-feeders might be on the basis of this functional relapse, and is an important limitation to take into consideration during prolonged enrichment of microbial communities. As previously described in other microbiome selection systems (38, 50, 51), the artificial selection or enrichment of microbial communities for a specific trait might cause the presence of so-called cheater microorganisms, that, in this case, benefit from solubilized P generated by other microorganisms.

Bacterial consortia capable of relieving plant P-stress proved to be rich in bacterial isolates able to solubilize P *in vitro*. In order to evaluate whether single isolates, hallmarked by specific *in vitro* characteristics, could improve plant growth promotion and P-uptake in maize, a deliberately chosen diverse subset of isolates was evaluated for their P-effect in plants. Although it is more likely that an isolate that performs well *in vitro* also has a positive effect *in planta*, our results show that the lack of performance *in vitro* does not automatically guarantee the lack of performance *in planta* (i.e., isolates I.115, I.178, I.071, and I.089). In this study, isolate I.115, which was identified as *Rhizobium* sp., was picked out as an extreme example hereof. It had an overall low *in vitro* ranking of 243/249, but was able to improve both plant growth and P-uptake. The beneficial effect of a bacterial strain is not only dependent on its *in vitro* performance, but can also be of indirect nature, such as changing the microbial community structure, changing the plant root architecture or the production of plant hormones (52–59). Finally, in a natural environment, arbuscular mycorrhizae (AMF) play an important role in the interplay between plants and bacteria. Apart from providing an extension to the plant root system, AMF can provide a niche for other beneficial microorganisms (60). On the other hand, mycorrhizae helper bacteria (MHB) can promote the development and functioning of AMF by triggering diverse plant growth factors (61). Among these MHB, *Burkholderia* spp., Enterobacter spp., Pseudomonas spp., and *Bacillus* spp. are some well-known examples that were also retrieved in our study.

An important requirement for the *in planta* efficiency of a strain is its ability to colonize the rhizosphere. In this research, we used a simple plating technique, followed by colony PCR targeting the 16S rRNA gene and Sanger sequencing to evaluate the presence of the bacterial genera at the end of the experiment. Isolates I.026, I.182, I.157, and I.034, which were all identified as *Pantoea* spp. and had a positive effect on plant growth and P-uptake, were all picked up in the rhizosphere of the inoculated maize plants at the end of the experiment. On the other hand, isolates I.115 and I.178, which were identified as *Rhizobium* sp. and *Burkholderia* sp., also proved to have a positive effect on maize, but were not picked up in the rhizosphere at the end of the experiment. This might be due to the slow growing nature of these isolates on microbial growth media rather than the inability to colonize the rhizosphere, since the selection of bacterial colonies was done after 24 h growth (62). Finally, isolates belonging to the genera *Pantoea* and Pseudomonas had a differential P-solubilizing and growth-promoting effect within both *in vitro* and *in planta* experiments (Fig. 4c and Fig. 5b), confirming the well documented, versatile nature of both genera in our results ([63] and references therein, [64, 65]).

To gain insight in the key mechanisms that render a bacterium capable of alleviating P-stress in plants, the *in vitro* characteristics of the bacterial isolates were linked to their phylogenetic distribution. Specific mechanisms of action (i.e., organic acid production and siderophore production) proved to be taxonomically well-structured traits. This observation substantiates previous findings concerning the phylogenetic distribution of siderophore producing bacteria (66). The ability of a bacterium to use different insoluble P-sources (i.e., FeP, AlP, and TCP) for their growth appeared to be unrelated to its phylogeny and was more dispersed in the phylogenetic cladogram (Fig. 4c). Since the ability to use a specific P-source is directly linked to specific mechanisms of actions (i.e., siderophore production, acid production, etc.), the complexity of this trait is less straightforward. The fact that the dispersion of a trait between different clades is correlated with its complexity substantiates our findings (67). Altogether, isolates that displayed an overall high performance *in vitro* (i.e., top 50 isolates) clustered together in the phylogenetic cladogram.

### Conclusion.

Isolates originating from a bacterial suspension that was able to relieve P-stress in maize were prominently present among the best performing isolates *in vitro*. These results substantiate the effectiveness of the holistic *in planta*-based enrichment and selection technique for the isolation of functional PSB. The commonly adopted isolation strategy generally consists of an *in vitro* isolation, screening and selection procedure of numerous cultured isolates on microbiological media in order to assess the desired plant growth-promoting trait, after which only a handful are being challenged in actual interaction with the host plant rhizosphere. In this case, *in vitro* selection takes precedence over *in planta* selection, which is after all the hallmark for PSB effectiveness. For future research and development, we suggest a more ecologically relevant approach for the selection of PSB, starting off with an *in planta* enrichment of rhizosphere competent phosphate solubilizing consortia. Secondly, as cultivability remains an important trait toward production and application as biostimulant, the cultivable fraction of these consortia can be isolated in a second selection step. Thus, ff *in vitro* trials are required to reduce the number of potential candidates for further greenhouse of field trials, these isolates should then be subjected to testing of well-considered parameters for the *in vitro* evaluation of P-solubilizing traits, which should be based on (i) the origin of the consortia and (ii) the intended use of the bacteria. In this approach, the primordial selection pressure for PSB initially takes place in the plant environment, and only then in the lab environment.

## MATERIALS AND METHODS

### Bacterial strains and culture conditions.

Bacterial strains were isolated from a rhizosphere start suspension and from mixed bacterial suspensions obtained through *in planta* enrichment for PSB (38). In short, a rhizosphere start suspension (RSS) was obtained by sampling the maize rhizosphere from different locations in Flanders. Subsequently, the RSS was used in an *in planta* enrichment concept, passed 4 enrichments after which respectively 4 mixed bacterial suspensions were obtained (V-B1, V-B2, V-B3, and V-B4) with 6 biological replicates each. The RSS and bacterial suspensions were plated on National Botanical Research Institute’s Phosphate (NBRIP) growth-agar supplemented with iron- and aluminum phosphate as P-sources (27) (Table S1). After 5 days of growth, 250 bacterial colonies were randomly selected from the plates (10 per biological replicate; RSS: 10, V-B1: 60, V-B2: 60, V-B3: 60, V-B4: 60) and transferred to LB-agar (per L: tryptone, 10 g; yeast extract, 5 g; NaCl, 10 g; bacteriological agar, 15 g) through streak-inoculation to obtain pure cultures. Isolates were grown in LB-medium (per L: tryptone, 10 g; yeast extract, 5 g; NaCl, 10 g) and stored in glycerol-stocks (23% glycerol) at −80°C.

### Phosphate solubilization assays.

**(i) Categorical phosphate solubilization assay.** The bacterial isolates were tested for their ability to grow on solid medium with insoluble phosphorus. As a growth medium, we used NBRIP-agar supplemented with either tri-calcium phosphate (TCP, Ca_3_(PO_4_)_2_), aluminum phosphate (AlPO_4_), or iron phosphate (FePO_4_.2H_2_O) (Table S1). Bacterial isolates were transferred from LB-agar to NBRIP-agar by point inoculation and were incubated for 48 h (TCP) to 5 days (FePO_4_.2H_2_O and AlPO_4_), after which colony and halo diameters were evaluated.

**(ii) Quantitative phosphate solubilization assay.** The phosphate solubilizing capacity of the bacterial isolates was evaluated quantitatively in liquid NBRIP-medium supplemented with a combination of iron- and aluminum phosphate in a 1:1 mol ratio (Table S1). Overnight grown cultures in LB-broth were washed in phosphate-buffered saline and subsequently inoculated in NBRIP-medium in a 1:10-ratio (v:v). Bacteria were incubated for 5 days (21°C, 140 rpm), after which samples were collected and centrifuged (5000 rpm, 10 min) to separate bacteria and insoluble phosphorus (pellet) from soluble phosphates (supernatants). Soluble phosphates were determined by an adaptation of the colorimetric method of Fiske & Subbarow (1925) by adding 1.5% (w:v) ammonium molybdate (in a 5.5% (v:v) H_2_SO_4_ solution) to the samples in a 1:1 (v:v) ratio (68). The phosphomolybdate produced was measured spectrophotometrically at 400 nm against a standard curve prepared with K_2_HPO_4_ ranging from 0 to 40 mg P.L^−1^.

### Organic acid production assay.

The ability of bacterial isolates to produce organic acids was tested on modified Pikovskaya’s (MPVK) agar (69). The medium composed of (per L): glucose, 10 g; (NH_4_)_2_SO_4_, 0.5 g; NaCl, 0.2 g; MgSO_4_.7H_2_O, 0.1 g; MnSO_4_.7H_2_O, 0.002 g; FeSO_4_.7H_2_O, 0.002 g; KCl, 0.2 g; yeast extract, 0.5 g; FePO_4_.2H_2_O, 1.5 g; AlPO_4_, 1 g; bacteriological agar, 30 g; 6 mL 0.4% (w:v) bromophenol blue (in technical ethanol), and was set at pH 6.7. Bacterial isolates were transferred from LB-agar, point inoculated on MPVK-agar and incubated for 48 h at room temperature, after which halo diameters were measured.

### Siderophores assay.

Bacterial siderophores production was evaluated on chrome azurol S (CAS) medium (70). All glassware was treated with 6M HCl prior to use to remove iron remnants. First, the dye solution was prepared by dissolving 60.5 mg chrome azurol S in 50 mL distilled water, adding 10 mL iron(III)-solution (1 mM FeCl_3_.6H_2_O in 10 mM HCl), and finally mixing the solution with a solution of 72.9 mg hexadecyltrimethyl-ammonium bromide (CTAB) in 40 mL distilled water. The Minimal Medium 9 (MM9) solution composed of (per 500 mL): KH_2_PO_4_, 15 g; NaCl, 25 g and NH_4_Cl, 50 g. A 20%-glucose solution was prepared by adding 20 g glucose to 100 mL distilled water. A casamino acid solution was prepared by adding 3 g Casamino Acids (Merck) to 27 mL distilled water, after which trace iron was removed through extraction with 3% (wt/vol) 8-hydroxyquinolin in chloroform and finally the solution was filter sterilized. A PIPES-solution was prepared composing of (per 750 mL): agar (bacteriological), 15 g; 1,4-piperazinediethanesulfonic acid (PIPES), 30.24 g; pH 6.8 with NaOH. To prepare the CAS-medium, 750 mL of PIPES-solution was mixed with 100 mL MM9, 10 mL 20%-glucose solution and 30 mL casamino acid solution. Finally, 100 mL of the dye solution was gently added to the agar. Single colonies were transferred from KB-agar (Labconsult) and streak-inoculated on CAS-agar. Plates were incubated at 21°C and evaluated after 5 days.

### H_2_S-production assay.

Bacterial hydrogen sulfide production was evaluated on sulfide indole motility (SIM) medium (Merck). Single colonies were transferred from LB-agar and were stab-inoculated in triplicate in 96-deepwell plates filled with SIM-medium. Plates were incubated at 26°C and evaluated after 48 h.

### Characterization of the isolates.

Bacterial isolates were characterized to the genus level by partial sequencing of the 16S rRNA gene. Genomic DNA was extracted through a colony PCR procedure. Single colonies of overnight grown cultures were suspended in 50 μL nuclease free water, incubated at 95°C for 10 min and centrifuged at 15.000 g for 5 min. The supernatants were subjected to PCR analysis. In case of failed amplification, samples were freeze-thawed and the above-mentioned extraction procedure was repeated. Samples for which the colony PCR procedure was inadequate were reanalyzed using the DNeasy UltraClean Microbial Kit (Qiagen) and genomic DNA was subjected to PCR. The bacterial V1-V9 regions of the 16S rRNA were amplified using the universal 27f (5′-AGAGTTTGATCCTGGCTCAG-3′) and 1492r (5′-GGTTACCTTGTTACGACTT-3′) primer pair (71). For the PCR MasterMix, the GoTaq G2 polymerase (Promega Benelux) was used according to the manufacturer’s specifications at a total volume of 20 μL per reaction. PCR conditions were as follows: initial denaturation at 94°C for 2 min, followed by 30 cycles of denaturation at 95°C for 20 s, annealing at 50°C for 20 s and elongation at 72°C for 1 min 30 s. A final elongation was carried out at 72°C for 5 min. The PCR product was analyzed on a 1% agarose gel (120 V, 30 min) and purified using the E.Z.N.A. MicroElute Cycle Pure-kit (Omega bio-tek, VWR). Finally, the samples were sequenced by LGC genomics GmbH, further processed using BioEdit (version 7.2), and the obtained sequences were identified using the BLAST tool on NCBI.

### Phylogenetic cladogram construction.

The obtained contigs from the forward- and reverse sequences were aligned using ClustalW multiple alignment in BioEdit (version 7.2). The phylogenetic cladogram was constructed using MEGA11 (72) and annotated using the iTOL software (73).

### Plant material and growth substrate.

Untreated maize (Zea mays L.) seeds (hybrid var. LG 30.217; Limagrain) were pregerminated in vermiculite (Vermex M; Soprema) for 7 days at 21°C. Seedling roots were rinsed with tap water and transplanted to 1 L pots containing 665 ± 5 g of growth substrate. As growth substrate, a mixture of washed, non-sterile fine quartz sand (Vosschemie Benelux) and a water-absorbent synthetic polymer (DCM Aquaperla) was used. The polymer was added to distilled water in a 1:100 (w:v) ratio, set to form a gel for 2 h and was subsequently mixed with fine sand in a 1.5:10 (v:w) ratio (74). A mixture of iron- and aluminum phosphate (FePO_4_.2H_2_O and AlPO_4_, respectively) was used in a 1:1 mol-ratio at a final concentration of 40 mg P per recipient and was added to the polymer mixture under constant stirring for homogenous distribution.

### *In planta* evaluation of bacterial isolates.

Plants were inoculated with bacterial suspensions by means of liquid application in 6 replicates. In short, overnight grown cultures in Tryptic Soy Broth (Merck) were washed in Phosphate Buffered Saline (PBS) and set at an optical density (OD_600_) of 0.7 ± 0.1. Subsequently, 1 mL of the liquid inoculum (or sterile PBS in case of the uninoculated control) was added within 1 cm of the maize seedling per treatment (or uninoculated control). Pots were incubated in a controlled environment for 21 days (T 21°C, RH 40%, 255.5 μmol.m^−2^.s^−1^ photosynthetically active radiation [PAR], light: dark 16 h: 8 h) under sun LED modules (SLMs; Phenovation), and were irrigated with a modified Hoagland solution (Table S2) (75, 76). At the end of the experiment, plant height, number of leaves and shoot- and root fresh weights were evaluated. Plant material was dried for 48 h at 60°C, after which shoot- and root dry weights were evaluated. Finally, shoot P-content was measured by means of ICP-OES as previously described in De Zutter et al. (38).

### Data processing and statistical analysis.

Shifts in frequency distributions of different bacterial traits (i.e., growth on NBRIP-agar with different P-sources, OA production, and siderophore production) between the different bacterial consortia (i.e., B-V1, B-V2, B-V3, and B-V4) were statistically evaluated using a Chi-squared test of independence in SPSS (IBM SPSS statistics, version 25.0). In order to have a valid comparison, the RSS, which was used as the starting point for *in planta* enrichment of PSB, was not included in the statistical analysis. Due to the biologically different nature of the RSS, which was not subjected to cyclic enrichment, this consortium was excluded from statistical analysis.

In order to detect structural regularities between bacterial isolates and possible associations between both numeric and nominal variables, the data were explored by means of a factor analysis of mixed data (FAMD) combined with hierarchical clustering on principal components (HCPC). Analysis of these data was performed in RStudio version 3.5.1 (77) using the functions ‘*FAMD’* and ‘*HCPC’* from the R-package ‘*FactoMineR’* (78). Interpretation of the model was restricted to the first 2 dimensions (46.5% of explained variance) based on the visual inspection of the scree plots using the function ‘*fviz_screeplot’* in the R-package *‘factoextra’* (Fig. S4) (79).

### Data availability.

All data sets were made available for review as supplemental data sets and will be available for public upon request. The sequences from BLAST analysis of partial 16S rRNA gene sequences were submitted to the NCBI database under accession number OP435374 to OP435581.
