# Supplementary material for: Innovative Rhizosphere-Based Enrichment under P-Limitation Selects for Bacterial Isolates with High-Performance P-Solubilizing Traits
Source: Microbiol Spectr. 2022 Oct 11;10(6):e02052-22. doi: 10.1128/spectrum.02052-22 (PMC9769856; doi:10.1128/spectrum.02052-22)
Supplement: Supplemental file 1 — Supplemental material. Download spectrum.02052-22-s0001.pdf, PDF file, 0.5 MB [file spectrum.02052-22-s0001.pdf]

Microbiology Spectrum Supplementary files

Article title: Innovative rhizosphere-based enrichment under P-limitation selects for bacterial isolates with high-performance P-solubilizing traits

Authors: Noémie De Zutter, Maarten Ameye, Pieter Vermeir, Jan Verwaeren, Leen De Gelder, Kris Audenaert

Supplementary Tables

Table S1 Composition of National Botanical Research Institute's Phosphate (NBRIP) growth medium

Medium	Composition	Reference
National Botanical Research Institute's Phosphate (NBRIP) growth medium (solid)	Per litre: glucose, 10.00 g; $(NH_4)_2SO_4$, 0.10 g; $MgSO_4.7H_2O$, 0.25 g; KCl, 0.20 g; $MgCl_2.6H_2O$, 5.00 g; P-source*:<br>• $Ca_3(PO_4)_2$ 5 g + 15 g agar<br>• $FePO_4.2H_2O$ 3.02 g + 25 g agar<br>• $AlPO_4$ 1.97 g + 25 g agar<br>• Combination of $FePO_4.2H_2O$ and $AlPO_4$ 1.51 g + 0.987 g + 25 g agar	Nautiyal, 1999
National Botanical Research Institute's Phosphate (NBRIP) growth medium (liquid)	Per litre: glucose, 10.00 g; $(NH_4)_2SO_4$, 0.10 g; $MgSO_4.7H_2O$, 0.25 g; KCl, 0.20 g; $MgCl_2.6H_2O$, 5.00 g; P-source (1 g $P.L^{-1}$): $FePO_4.2H_2O$, 3.02 g; $AlPO_4$, 1.97 g (1:1 mole ratio)	Nautiyal, 1999

*Based on the experimental ability of the agar to solidify

Table S2 Composition of the modified Hoagland's solution used in the plant experiments (Hoagland & Arnon, 1950; Cooper & Burton, 2004).

Name	Formula	Grams per litre	Millilitres of stock solution per litre of nutrient solution
Calcium nitrate, tetra hydrate	$Ca(NO_3)_2.4H_2O$	236.15	4
Potassium nitrate	KNO_3	101.11	6
Magnesium sulfate, hepta hydrate	$MgSO_4.7H_2O$	246.36	2
Iron EDTA	$C_{10}H_{12}FeN_2NaO_8.3H_2O$	21.11	1
Micronutrients*			1

*Contains (per litre): H_3BO_4, 2.86 g; $MnCl_2.2H_2O$, 1.47 g; $ZnSO_4.7H_2O$, 0.22 g; $CuSO_4.5H_2O$, 0.08 g; $(NH_4)_6Mo_7O_{24}.4H_2O$, 0.018 g.

Table S3 Comparison between frequency distributions of various classes of siderophore production in the different consortia. Values represent p-values as per Chi-squared test of independence.

	RSS	B-V1	B-V2	B-V3	B-V4
RSS	-	0.078	<0.001	0.118	0.660
B-V1		-	<0.001	<0.001	0.018
B-V2			-	<0.001	<0.001
B-V3				-	<0.001
B-V4					-

Table S4 Top 50 ranking of the bacterial isolates based on the combined score of numeric (growth on NBRIP-agar supplemented with either iron-phosphate (FeP), aluminium phosphate (AlP) or tri-calcium phosphate (TCP); P-solubilizing capacity in liquid NBRIP supplemented with both iron- and aluminium phosphate) and nominal (organic acid production on MPVK-agar and siderophore production on CAS-agar) traits.

Isolate	Consortium	FeP	AlP	TCP	Dissolved P	OA	Siderophores	Total score	Rank
I.026	B-V1	0.78	0.71	0.83	0.80	1.00	0.67	4.78	1
I.028	B-V1	0.78	0.65	0.77	0.84	1.00	0.67	4.69	2
I.059	B-V1	1.00	0.65	0.47	0.85	1.00	0.67	4.63	3
I.218	B-V4	0.78	0.53	0.57	0.64	1.00	1.00	4.51	4
I.145	B-V3	0.67	0.53	0.30	1.00	1.00	1.00	4.50	5
I.149	B-V3	0.67	0.53	0.40	0.85	1.00	1.00	4.45	6
I.250	B-V4	1.00	0.71	0.70	0.00	1.00	1.00	4.41	7
I.186	B-V3	0.67	0.71	1.00	0.00	1.00	1.00	4.37	8
I.212	B-V4	0.67	0.53	0.50	0.64	1.00	1.00	4.34	9
I.138	B-V3	0.89	0.53	0.73	0.00	1.00	1.00	4.15	10
I.154	B-V3	0.78	0.71	0.60	0.06	1.00	1.00	4.15	11
I.142	B-V3	0.67	0.53	0.30	0.64	1.00	1.00	4.14	12
I.072	B-V2	0.78	0.59	0.57	0.48	1.00	0.67	4.08	13
I.181	B-V3	0.67	0.71	0.67	0.02	1.00	1.00	4.06	14
I.222	B-V4	0.67	0.71	0.67	0.01	1.00	1.00	4.05	15
I.190	B-V3	0.67	0.71	0.67	0.00	1.00	1.00	4.04	16
I.160	B-V3	0.67	0.71	0.63	0.02	1.00	1.00	4.02	17
I.188	B-V3	0.67	0.71	0.63	0.00	1.00	1.00	4.01	18
I.029	B-V1	0.78	0.65	0.87	0.02	1.00	0.67	3.98	19
I.185	B-V3	0.67	0.71	0.60	0.00	1.00	1.00	3.97	20
I.211	B-V4	0.33	0.53	0.53	0.58	1.00	1.00	3.97	21
I.182	B-V3	0.67	0.71	0.57	0.02	1.00	1.00	3.96	22
I.224	B-V4	0.44	0.88	0.60	0.02	1.00	1.00	3.94	23
I.189	B-V3	0.67	0.71	0.57	0.00	1.00	1.00	3.94	24
I.184	B-V3	0.67	0.71	0.50	0.05	1.00	1.00	3.92	25
I.183	B-V3	0.67	0.65	0.57	0.03	1.00	1.00	3.91	26
I.150	B-V3	0.67	0.53	0.40	0.30	1.00	1.00	3.90	27
I.146	B-V3	0.67	0.53	0.67	0.00	1.00	1.00	3.87	28
I.166	B-V3	0.67	0.65	0.53	0.00	1.00	1.00	3.85	29
I.219	B-V4	0.67	0.53	0.47	0.18	1.00	1.00	3.84	30
I.216	B-V4	0.78	0.53	0.53	0.00	1.00	1.00	3.84	31
I.080	B-V2	0.78	0.65	0.73	0.01	1.00	0.67	3.84	32
I.214	B-V4	0.67	0.59	0.57	0.00	1.00	1.00	3.82	33
I.217	B-V4	0.67	0.47	0.63	0.00	1.00	1.00	3.77	34
I.147	B-V3	0.67	0.59	0.50	0.00	1.00	1.00	3.76	35
I.243	B-V4	0.67	0.82	0.60	0.00	1.00	0.67	3.76	36
I.220	B-V4	0.67	0.53	0.50	0.01	1.00	1.00	3.71	37
I.213	B-V4	0.67	0.53	0.50	0.00	1.00	1.00	3.70	38
I.240	B-V4	0.67	0.53	0.50	0.00	1.00	1.00	3.70	39
I.231	B-V4	0.44	0.65	0.60	0.00	1.00	1.00	3.69	40
I.132	B-V3	0.67	0.53	0.47	0.00	1.00	1.00	3.66	41
I.144	B-V3	0.67	0.59	0.40	0.00	1.00	1.00	3.65	42
I.074	B-V2	0.67	0.71	0.57	0.02	1.00	0.67	3.63	43
I.164	B-V3	0.67	0.59	0.33	0.02	1.00	1.00	3.61	44
I.133	B-V3	0.78	0.53	0.30	0.00	1.00	1.00	3.61	45
I.141	B-V3	0.67	0.53	0.40	0.01	1.00	1.00	3.60	46
I.148	B-V3	0.67	0.53	0.40	0.00	1.00	1.00	3.60	47
I.163	B-V3	0.67	0.53	0.40	0.00	1.00	1.00	3.60	48
I.215	B-V4	0.67	0.53	0.40	0.00	1.00	1.00	3.60	49
I.044	B-V1	0.78	0.53	0.20	0.07	1.00	1.00	3.58	50

Table S5 Description of each cluster by numeric variables after hierarchical clustering on principal components.

		v.test	Mean category	Overall mean	s.d. mean	Overall s.d.	p.value
Cluster 1	TCP	-4.89	2.04	2.91	0.79	1.76	<0.001
	AlP	-5.84	2.38	2.87	0.72	0.83	<0.001
	FeP	-8.23	0.90	1.60	0.82	0.83	<0.001
Cluster 2	AlP	-4.54	2.43	2.87	0.60	0.83	<0.001
	TCP	-5.60	1.75	2.91	1.02	1.76	<0.001
Cluster 3	NULL						
Cluster 4	AlP	8.40	3.5	2.87	0.62	0.83	<0.001
	TCP	7.24	4.07	2.91	1.71	1.76	<0.001
	FeP	5.9	2.04	1.60	0.53	0.83	<0.001
	Dissolved P	-2.57	12.02	26.77	20.65	63.65	0.01
Cluster 5	Dissolved P	14.49	299.16	26.77	61.52	63.65	<0.001
	TCP	3.72	4.85	2.91	2.00	1.76	<0.001
	FeP	2.51	2.21	1.60	0.52	0.83	0.01

Table S6 Description of each cluster by categorical variables after hierarchical clustering on principal components.

		Cla/Mod	Mod/Cla	Global	p.value	v.test
Cluster 1	S Class 1	71.43	85.71	34.15	<0.001	10.77
	No OA production	64.00	68.57	30.49	<0.001	7.96
	S Class 2	0	0	12.60	<0.001	-4.35
	S Class 3	7.69	7.14	26.42	<0.001	-4.61
	No siderophore production	7.58	7.14	26.83	<0.001	-4.69
	OA production	12.87	31.43	69.51	<0.001	-7.96
Cluster 2	No siderophore production	84.85	100	26.83	<0.001	14.16
	S Class 2	0	0	12.60	<0.001	-3.75
	S Class 3	0	0	26.42	<0.001	-5.98
	S Class 1	0	0	34.15	<0.001	-7.10
Cluster 3	S Class 2	87.10	100	12.60	<0.001	11.79
	S Class 3	0	0	26.42	<0.001	-3.80
	No siderophore production	0	0	26.83	<0.001	-3.84
	S Class 1	0	0	34.15	<0.001	-4.54
Cluster 4	S Class 3	83.08	65.85	26.42	<0.001	9.81
	OA production	46.20	96.34	69.51	<0.001	7.07
	S Class 2	0	0	12.60	<0.001	-4.85
	No siderophore production	6.06	4.88	26.83	<0.001	-5.94
	No OA production	4.00	3.66	30.49	<0.001	-7.07
Cluster 5	OA production	6.43	100	69.51	0.017	2.40
	S Class 2	12.90	36.36	12.60	0.042	2.03
	S Class 3	9.23	54.54	26.42	0.050	1.96
	No OA production	0	0	30.49	0.017	-2.40
	S Class 1	0	0	34.15	0.009	-2.61

Table S7 Classification of bacterial isolates per group (RSS, n = 10; B-V1, n = 60; B-V2, n = 59; B-V3, n = 60; B-V4, n = 60) based on 16S rRNA at a genus level.

Phylum	Order	Genus	RSS	B-V1	B-V2	B-V3	B-V4
Actinobacteria	Actinomycetales	Curtobacterium	4	0	0	0	0
Firmicutes	Bacillales	Bacillus	1	1	2	2	0
	Bacillales	Paenibacillus	0	0	1	3	0
Others	Others	Yeast	0	7	9	12	0
Proteobacteria	Burkholderiales	Burkholderia-Paraburkholderia	0	0	2	2	0
	Enterobacterales	Enterobacter	0	1	2	0	0
		Enterobacter-Atlantibacter-Kosakonia	0	1	7	0	1
		Erwinia	0	0	2	1	3
		Klebsiella-Raoultella	2	0	0	0	0
		Leclercia	0	0	0	2	0
		Leclercia-Enterobacter	0	3	2	0	1
		Pantoea	2	12	16	36	25
		Rahnella-Ewignella	1	0	0	0	0
		Rouxiella	0	12	0	0	0
		Rouxiella-Rahnella	0	2	0	0	0
	Pseudomonadales	Pseudomonas	0	19	11	1	25
	Rhizobiales	Rhizobium-Agrobacterium	0	1	3	0	2
Uncultured bacterium	Uncultured bacterium	Uncultured bacterium	0	1	2	1	3

Table S8 Percentual increase/decrease of prevalence of bacterial orders per consortium, compared to B-V3.

	Actinomycetales	Bacillales	Burkholderiales	Enterobacterales	Pseudomonadales	Rhizobiales	Uncultured bacterium	Others
RSS	+40%	+1.67%	-3.33%	-15%	-1.67%	+0%	-1.67%	-20%
B-V1	+0%	-6.66%	-3.33%	-13.33%	+30%	+1.67%	+0%	-8.33%
B-V2	+0%	-3.25%	+0.06%	-15.85%	+16.97%	+5.08%	+1.72%	-4.75%
B-V3	(n = 0)	(n = 5)	(n = 2)	(n = 39)	(n = 1)	(n = 0)	(n = 1)	(n = 12)
B-V4	+0%	-8.33%	-3.33%	-15%	+40%	+3.33%	+3.33%	-20%

Table S9 Distribution of bacterial isolates (taxonomic level: order) in the different clusters obtained through HCPC. Cluster 1: n = 70; Cluster 2: n = 56; Cluster 3: n = 27; Cluster 4: n = 82; Cluster 5: n = 11.

		Cluster 1	Cluster 2	Cluster 3	Cluster 4	Cluster 5
Actinobacteria	Actinomycetales	4.3 %	0.0 %	0.0 %	1.2 %	0.0 %
Firmicutes	Bacillales	5.7 %	5.4 %	0.0 %	0.0 %	0.0 %
Proteobacteria	Burkholderiales	4.3 %	1.8 %	0.0 %	0.0 %	0.0 %
Proteobacteria	Enterobacterales	30.0 %	21.1 %	44.4 %	89.0 %	90.9 %
Proteobacteria	Pseudomonadales	41.4 %	12.5 %	55.6 %	6.1 %	0.0 %
Proteobacteria	Rhizobiales	8.6 %	0.0 %	0.0 %	0.0 %	0.0 %
Uncult. bacterium	Uncult. bacterium	4.3 %	3.6 %	0.0 %	2.4 %	0.0 %
Others	Others	1.4 %	44.6 %	0.0 %	1.2 %	9.1 %

Table S10 Prevalence of the inoculated bacterial genera in the rhizosphere of maize plants at the end of the plant experiments.

Isolate	ID	Colony PCR		Fraction equivalent
		Pass	Fail	to ID
I.026	*Pantoea sp.*	9	21	56 %
I.034	*Pantoea sp.*	3	27	33 %
I.037	*Pseudomonas sp.*	9	21	89 %
I.039	*Pseudomonas sp.*	18	12	100 %
I.040	*Yeast*	4	26	0 %
I.049	*Pantoea sp.*	3	27	0 %
I.059	*Pantoea sp.*	20	10	20 %
I.071	*Enterobacter sp.*	12	18	42 %
I.089	*Pseudomonas sp.*	10	20	100 %
I.090	*Pseudomonas sp.*	15	15	87 %
I.111	*Burkholderia sp.*	18	12	0 %
I.115	*Rhizobium sp.*	9	21	0 %
I.135	*Bacillus sp.*	20	10	0 %
I.157	*Pantoea sp.*	13	17	23 %
I.178	*Burkholderia sp.*	6	24	0 %
I.182	*Pantoea sp.*	15	15	33 %
I.246	*Pantoea sp.*	16	14	13 %

Supplementary Figures

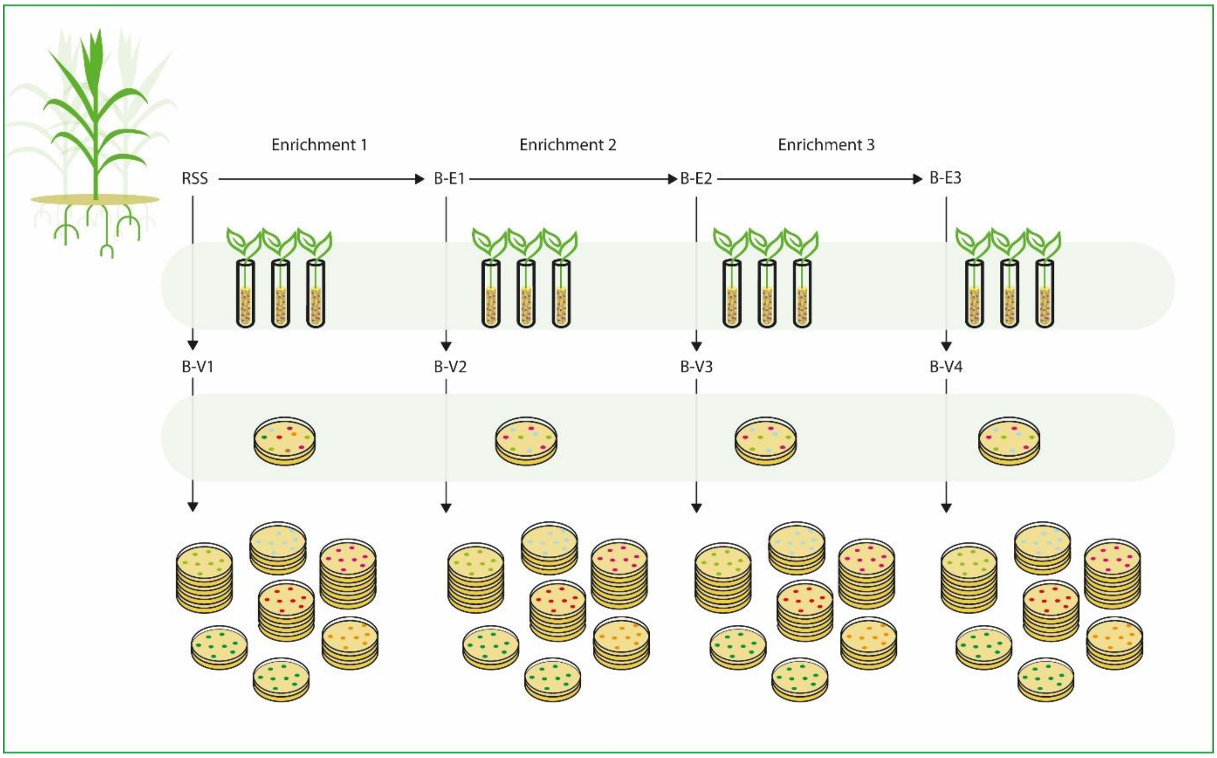

Figure S1 Overview of the origin of the enriched bacterial consortia (De Zutter et al. 2021), which were the source for the selection and screening of the bacterial isolates used in this research.

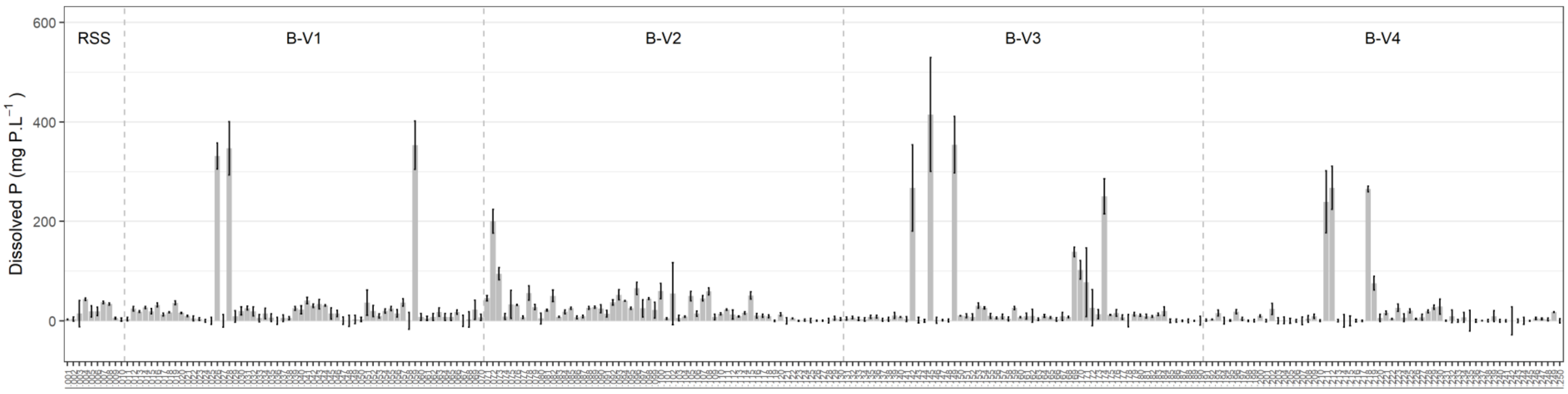

Figure S2 P-solubilizing capacity of bacterial isolates per group (RSS, B-V1, B-V2, B-V3, B-V4) in liquid NBRIP supplemented with iron- and aluminium phosphate. Values represent mean ± s.d. of three biological replicates.

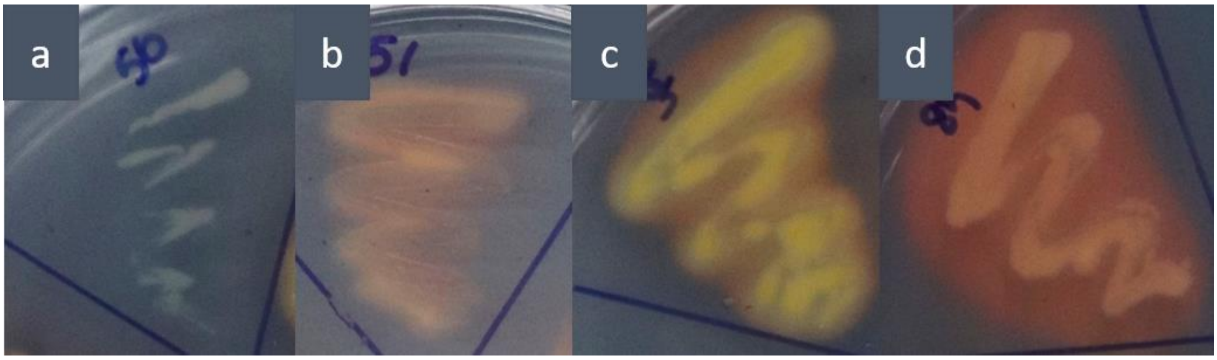

Figure S3 Visual classification of siderophore production into different classes. (a) No siderophore production, (b) Class 1, (c) Class 2, (d) Class 3.

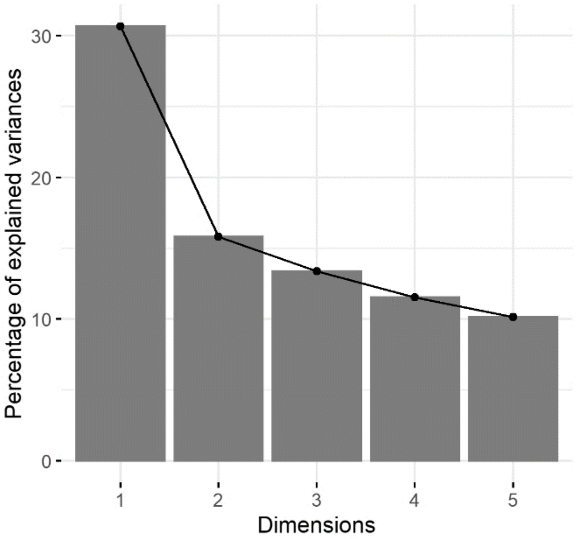

Figure S4 Scree plots of Factor Analysis of Mixed Data (FAMD). Based on numeric data (diameters on NBRIP supplemented with TCP, FeP and AlP; P-solubilizing capacity in liquid medium) and nominal data (organic acid production; siderophore production).
